# Hysteresis Can Grant Fitness in Stochastically Varying Environment

**DOI:** 10.1371/journal.pone.0103241

**Published:** 2014-07-28

**Authors:** Gary Friedman, Stephen McCarthy, Dmitrii Rachinskii

**Affiliations:** 1 Department of Electrical and Computer Engineering, Drexel University, Philadelphia, Pennsylvania, United States of America; 2 Department of Applied Mathematics, University College Cork, Cork, Ireland; 3 Department of Mathematical Sciences, University of Texas at Dallas, Richardson, Texas, United States of America; Baylor College of Medicine, United States of America

## Abstract

Although the existence of multiple stable phenotypes of living organisms enables random switching between phenotypes as well as non-random history dependent switching called hysteresis, only random switching has been considered in prior experimental and theoretical models of adaptation to variable environments. This work considers the possibility that hysteresis may also evolve together with random phenotype switching to maximize population growth. In addition to allowing the possibility that switching rates between different phenotypes may depend not only on a continuous environmental input variable, but also on the phenotype itself, the present work considers an opportunity cost of the switching events. This opportunity cost arises as a result of a lag phase experimentally observed after phenotype switching and stochastic behavior of the environmental input. It is shown that stochastic environmental variation results in maximal asymptotic growth rate when organisms display hysteresis for sufficiently slowly varying environmental input. At the same time, sinusoidal input does not cause evolution of memory suggesting that the connection between the lag phase, stochastic environmental variation and evolution of hysteresis is a result of a stochastic resonance type phenomenon.

## Introduction

Adaptation of organisms to time-varying and often uncertain environments is a classical problem in evolutionary biology. Existence of multiple phenotypes and random switching between them establishes phenotypic diversity within the population and has been suggested as a form of bet-hedging strategy that increases the chances of survival and growth rates of the total population [1]. It is intuitively clear that selection should favor those phenotype switching probabilities that match in some way environmental variation rates. To see what might be a good choice of the switching probabilities, Kussel & Leibler [2] compared random switching between phenotypes to responsive switching using a continuous time model for a discrete-valued environment by imposing a cost on the non-random responsive strategy and assuming that random switching rates are independent of the environment. Others [3] considered a different approach where switching probabilities were viewed as a single valued function of a binary environmental variable that may be favorable or unfavorable to a particular phenotype in terms of the phenotype growth rates. This work concluded that, under some circumstances, small switching probability from favorable to unfavorable phenotype may be advantageous for the growth of the entire population. In their experimental work [4]–[6] the same group was able to tune the phenotype switching probabilities utilizing bi-stability in the galactose utilization network of *Saccharomyces cerevisiae* obtaining agreement with the model.

**Figure 1:**
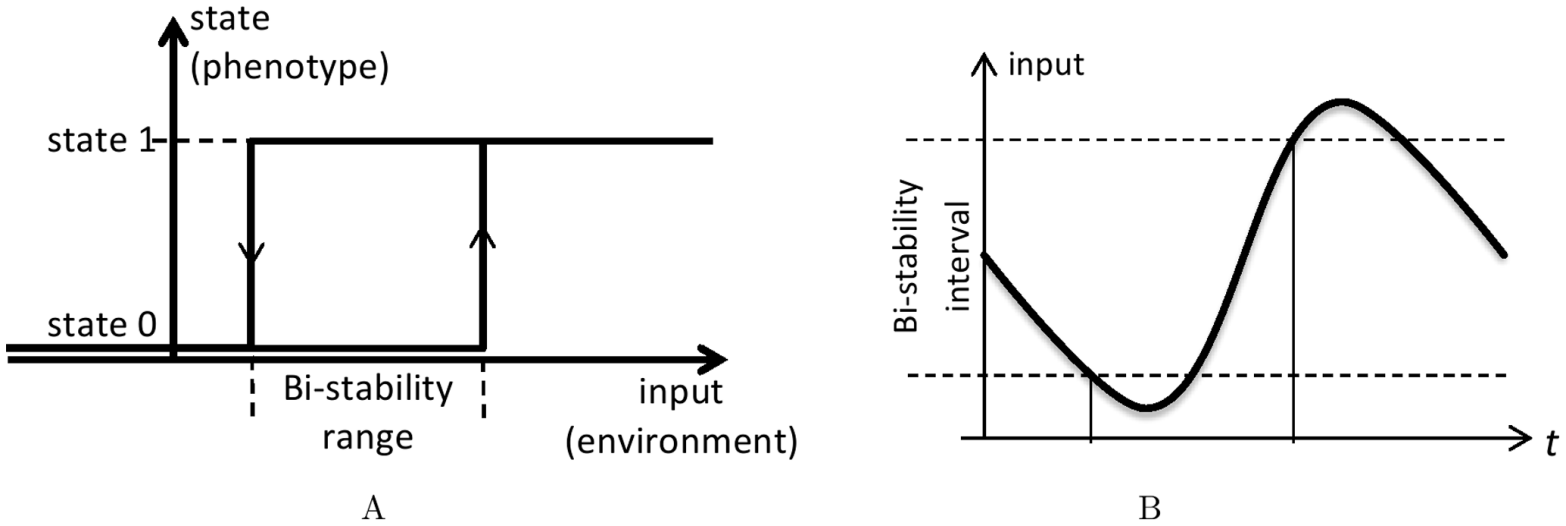
Illustration of bi-stable relay hysteresis. When the state is 1, the relay switches to state 0 at the lower input threshold, while starting from state 0 the relay switches to state 1 at an upper threshold. Thus, as long as the current input is within the bi-stability range (i.e. between the thresholds), the relay remembers whether the input has entered this range from below or from above. Panel A shows the input-state diagram. Panel B presents an example of input graph. The vertical interval between the horizontal dashed lines on panel B corresponds to the horizontal bi-stability interval on panel A. The intersection of the lower (upper) dashed line with the input graph on B defines a moment of switching from state 1 to 0 (from 0 to 1).

Phenotypic multi-stability in biological systems is related not only with bet-hedging behavior, but also with persistent memory of history called hysteresis. The term “hysteresis” seems to have been coined by James Alfred Ewing [7] in connection with the ability of some magnetic materials to retain their magnetization state long after the magnetizing magnetic field has been removed. Today it is used much more broadly to refer to any memory based relationship between an input and state of a system that does not depend on the rate at which the input varies in time [8]. The most basic, yet non-trivial, hysteresis is exemplified by a bi-stable relay illustrated in Figure 1, where the current state of the relay is determined not only by the external input, but also by the previous relay state. The key characteristic of the bi-stable relay hysteresis is the threshold separation, which is the difference between the values of the external input at which the state switches.

Classical example of a bi-stable relay hysteresis in biological systems is the history dependent behavior of the *lac-operon* studied in *E. coli* bacteria. *Lac-operon* can be viewed as a collection of genes associated with transport and metabolism of lactose. Novick & Weiner [9] as well as of Cohn & Horibata [10]–[12], relying on prior work of others [13]–[17], demonstrated that two phenotypes each associated with “on” and “off” state of the *lac-operon* expression can be obtained from the same culture of genetically identical bacteria. The fraction of each corresponding sub-population depended on the history of the exposure to the inducer. Similarly to the relay illustrated in Figure 1, the *lac-operon* state was induced (switched on) when the extracellular inducer concentration (input) exceeded an upper threshold. The operon was switched off when the inducer concentration fell below the lower threshold. Both phenotypes remain stable through multiple generations of the bacterial culture after the extracellular concentration of the inducer is reduced to lower levels, but not removed completely. Novick & Weiner did not use the term “hysteresis” to describe their observations, but effectively that is what it was.

**Figure 2:**
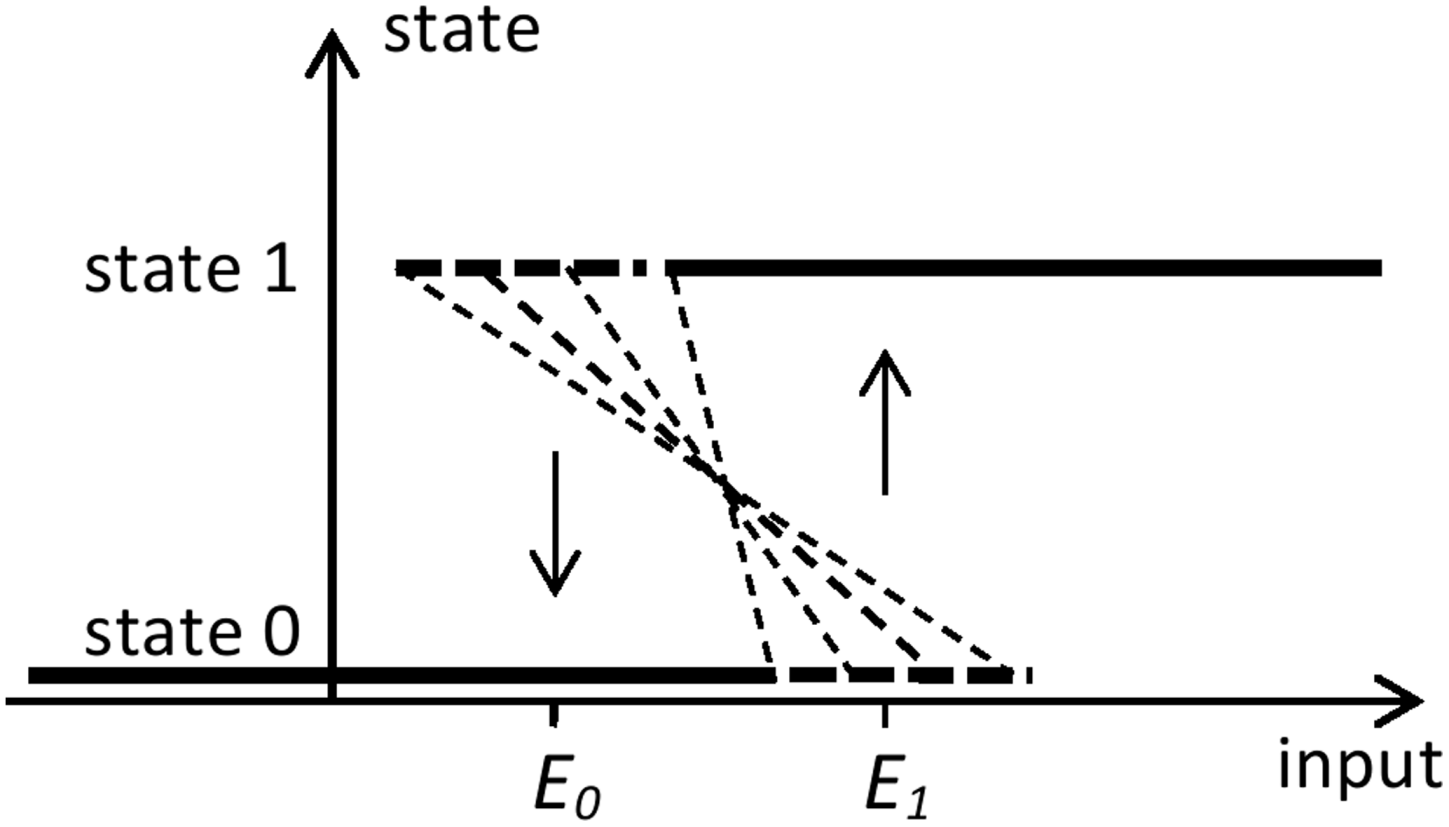
Illustration of random phenotype switching. The S-shaped curve consists of two horizontal segments and a slanted segment whose slope may be randomly changing. Stable phenotype states can be found on the horizontal segments of this curve. For a given value of the environmental input 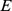, the transition from the state on the lower part of the curve to the upper part occurs when the meeting point of the slanted segment with the lower state line shifts to the left of 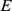; the transition from the upper to the lower branch occurs when the meeting point of the slanted segment with the upper state line shifts to the right of 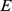. When 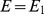, transitions from state 0 to state 1 occur with higher probability than transitions from state 1 to the state 0. When 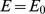, transitions from state 0 to state 1 are less likely than transitions from state 1 to state 0.

It has been pointed out a number of times that random switching and hysteretic switching (memory) can both be observed in biological systems having multiple stable phenotypes [18]–[32]. Consider, for example, bi-stable relay of the galactose utilization network of *Saccharomyces cerevisiae* studied in [6], [33] and used in experiments related to advantages of population diversity [34]. For a certain set of control parameters, the bi-stability displayed pronounced random switching between phenotypes. An illustration that does not rely on concepts related to thermodynamic equilibrium is shown in Figure 2. The figure shows an S-shaped curve whose features may be randomly changing due to random variations in gene expression, for example. Stable phenotype states can be found on the horizontal segments of this curve, while the segment with the negative slope is unstable. If one considers a binary environmental variable, the random changes in the curve features may cause random transitions from one stable state to another with different probabilities at different values of the binary environment. When the value of the environmental variable is 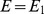, the transition from the state on the lower part of the curve to the upper part occurs with greater probability. On the other hand, when 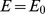, transitions from the upper to the lower branch are more likely. Such transitions may relatively quickly randomize the phenotype erasing any memory of the past phenotype state [35]–[41].

In the above description, one may treat the transition probability solely as a function of the environment because the environment is binary valued. This becomes more obvious for a different set of the regulatory network parameters when the galactose utilization network of *Saccharomyces cerevisiae* exhibits persistent memory over long period of time [6]. Random switching with rates similar to the previous case would occur in this case too, however, only when the input is sufficiently close to one of the two thresholds associated with the hysteresis. Therefore, both memory and random switching of phenotype can be observable if a continuum of the environmental input states was considered, some far from the thresholds and some close. Hence, if one wants to investigate the possibility and effects of hysteretic memory, one should assume the dependence of the transitions probabilities on the phenotype as well as on the value of the continuous environmental variable. This is exactly the approach taken in the present work. Thus, while the previous work focused on the random phenotype switching showing that appropriate choice of switching probabilities is advantageous in differently varying binary environments, in this work we focus on the possibility that hysteresis and memory can provide certain advantages in population growth by considering continuously varying environmental input. Rephrasing, the previous analysis dealt with the following optimization problem: given a certain switching rate to the favored phenotype for every possible state of the environment, find the value of the lower (typically small) transition rate to the unfavored phenotype that would maximize the growth of the total population. In this work, we are solving a different optimization problem by allowing a range of environmental states (between the thresholds) within which both switching rates to and from the favored phenotype are small (possibly zero) and using this range as an optimization parameter to maximize the growth rate.

That is, the question we ask is: how strong of a hysteresis effect may one expect to evolve? More precisely, what would be the most advantageous threshold separation and how is it affected by the variability and uncertainty of the environmental input?

This problem is studied below using a hybrid system model very similar to one used to describe population diversification in the experiments with *Saccharomyces cerevisiae* [3], [4]. However, the proposed model differs in two significant ways from the previous model.

One difference is that the proposed model explicitly introduces a non-growing phenotypes in addition to the two phenotypes capable of some growth in any environment being considered. The non-growing phenotypes are introduced instead of explicitly imposing a time delay as a model for lag phase in the growth of phenotypes after each switching event. On the one hand, using a non-growing phenotype is a self-consistent approach as the growth of each phenotype is not explicitly dependent on time. On the other hand, this approach simplifies the mathematical treatment by adding two random differential equations rather than using a smaller number of random differential equations with time delay.

The key difference between the previous and the proposed model is the possibility that switching rates between phenotypes depend not only on the environment, but also on the phenotype itself. This effectively implies that environmental input values (thresholds) at which different phenotypes change their switching rates are permitted to be different in the proposed model and is exactly what allows hysteretic memory to exist as a possible solution to the growth optimization problem. This optimization mechanism is only possible if the environment admits values between the thresholds, which is part of the reason that we model the variations of the environment by a continuous process.

With these modifications the growth model becomes: 
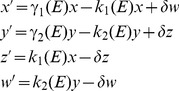
(1)where “prime” denotes time derivative; 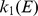 and 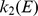 are environment dependent rates at which organisms switch from phenotypes 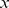 and 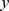, respectively, while 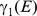 and 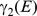 are the corresponding phenotype growth rates; 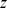 and 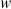 represent non-growing phenotypes; and, 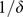 is the lag phase characteristic time.

Different functional dependencies of the coefficients in model (3) can be considered. Continuous functions (piecewise linear sigmoidal) are used here to model dependence of growth coefficients on the environment: 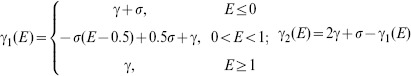
(2)where 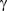 is the minimum growth rate possible and 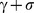 is the maximum possible growth rate. Effectively, 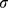 is the maximal growth rate advantage of the alternate phenotype.

Although the above dependence is employed here primarily to illustrate key features of the model, it can be viewed as reasonable because 1) the growth rates of both phenotypes can be the same at some value of the environmental variable (which is set to 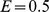 here) and 2) only a partial favoring of one phenotype over the other is possible when the environmental variable deviates from 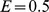 by a small amount. On the other hand, at large deviations of the environmental variable from its average, the difference in favoring one phenotype over another is bounded by some value 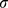.

Similarly to the previously considered models, the dependence of the switching rates 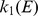, and 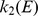 on the environmental variable will be described by step functions. However, in contrast to the previous model, the thresholds for these steps will not be required to be the same for the two growing phenotypes. We use a parameter 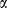 to specify the separation of thresholds: 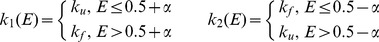
(3)where 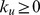 is the rate at which bacteria switch from favored to unfavored phenotype and 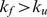 is the rate of switching from unfavored to favored phenotype. When 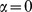, the change of the transition rate from one phenotype to another coincides with the change of the phenotype growth status from favored to unfavored or vice versa. This change of transition rates can be characterized as “realistic” strategy. Positive 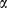 implies that there is an interval of the environmental input 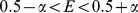 over which both growing phenotypes have low transition rates 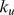. In this case one phenotype retains its low transition rate even after its growth status has changed to unfavored, while the other reduces its transition rate even before its growth status changes to favored. For this reason the strategy corresponding to positive 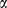 can be characterized as optimistic. Negative 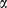 means that both phenotypes have high transition rate 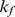 over the interval 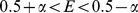 which corresponds to a pessimistic strategy, although somewhat different from bet-hedging.

Variation of the environment will be modeled here in two distinct ways: by a random process and by a periodic symmetric (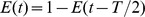) function. One of the random processes employed here is the Ornstein-Uhlenbeck (OU) process that describes diffusion-like motion in a one-dimensional parabolic potential centered at the point 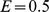 where the growth rates of the phenotypes are equal: 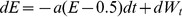
(4)where 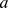 is a stiffness parameter associated with the parabolic potential well and 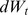 is the derivative of the Wiener process (white noise) creating stochastic fluctuations around the point 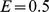. Average time 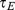 of passage of the interval 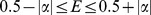 by the OU process can be viewed as a certain characteristic time of this process. A modification of the OU process that corresponds to a double-well, rather than a parabolic potential will also be considered: 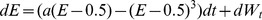
(5)


Deterministic periodic environmental variation will be taken as sinusoidal having a half-period 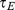.

The effect of the threshold 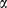 on the population growth will be investigated along with the effects of parameters 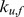 and 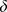 using the Lyapunov's exponent to represent the asymptotic growth: 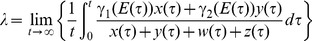
(6)


## Simulations

In order to obtain the dependence of the average growth rate (6) on the threshold separation distance 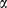 and other parameters, we changed variables and considered the population of cells in each phenotype in terms of its fraction of the total population 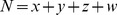. With the change of variables 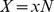, 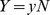, 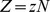, 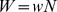, system (1) becomes 
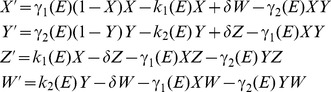
(7)


While populations in model (1) grow exponentially, solutions of model (7) fluctuate near a positive equilibrium.

**Figure 3:**
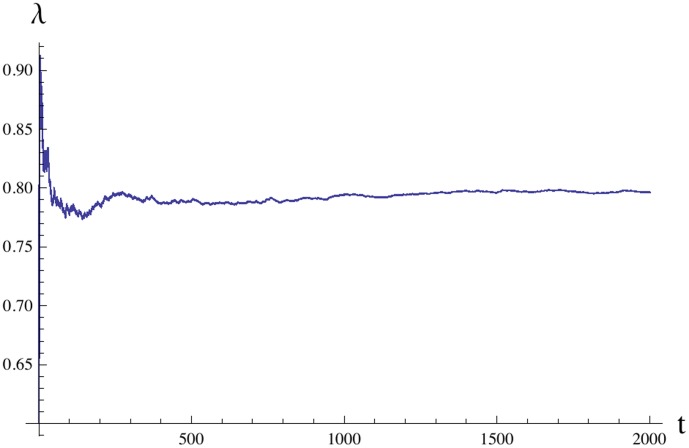
Convergence of the growth rate to the Lyapunov exponent. A typical evolution 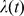 of the time average of the growth rate (8) over the increasing time interval from 0 to 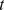. For 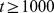 the process 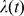 asymptotically approaches a stationary value (6). Deviations are within 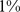. The plot was obtained using system (7) with OU input (4).

**Figure 4:**
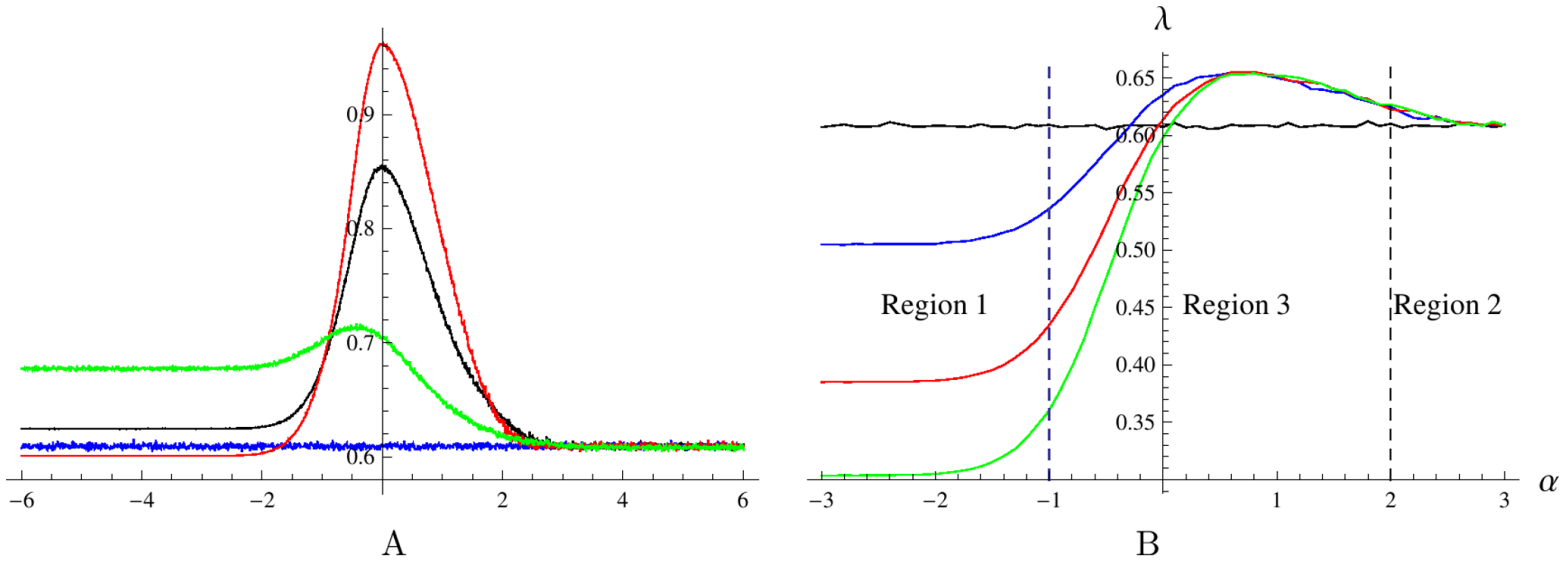
The Lyapunov's exponent 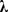 for different 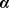 values. Plots on panels A and B were obtained for systems (9) and (1), respectively, with OU environmental input (4). A: The curves correspond to 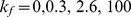. For 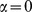 the growth rate 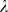 increases with 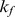. B: The curves are for 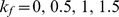. For negative 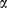 the growth rate 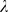 decreases with 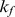. Other parameters are 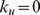, 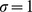, 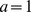, 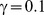, 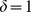.

**Figure 5:**
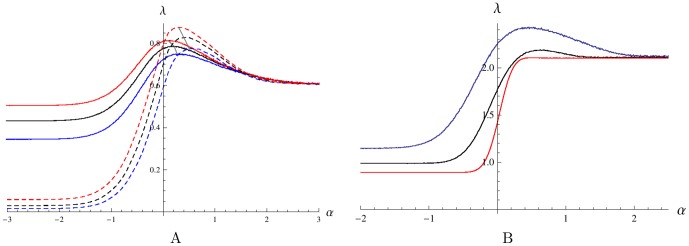
The effect of changes in the parameters 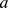 and 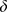 on the Lyapunov's exponent 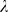. Each plot demonstrates a positive optimal value of the threshold parameter 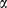, which maximizes 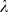 for model (1). A: The effect of altering the average lag time 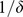. The curves correspond to 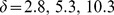. The growth rate 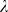 increases with 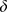. Solid lines are plotted for 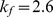, a relatively slow transition rate; dashed lines are plotted for 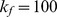, a high transition rate. Other parameters are 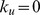, 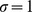, 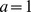 and 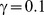. B: The effect of varying the stiffness of the potential well 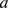. The lines correspond to 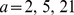. The growth rate 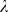 decreases with 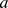. Other parameters are 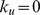, 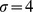, 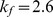, 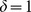 and 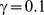.

**Figure 6:**
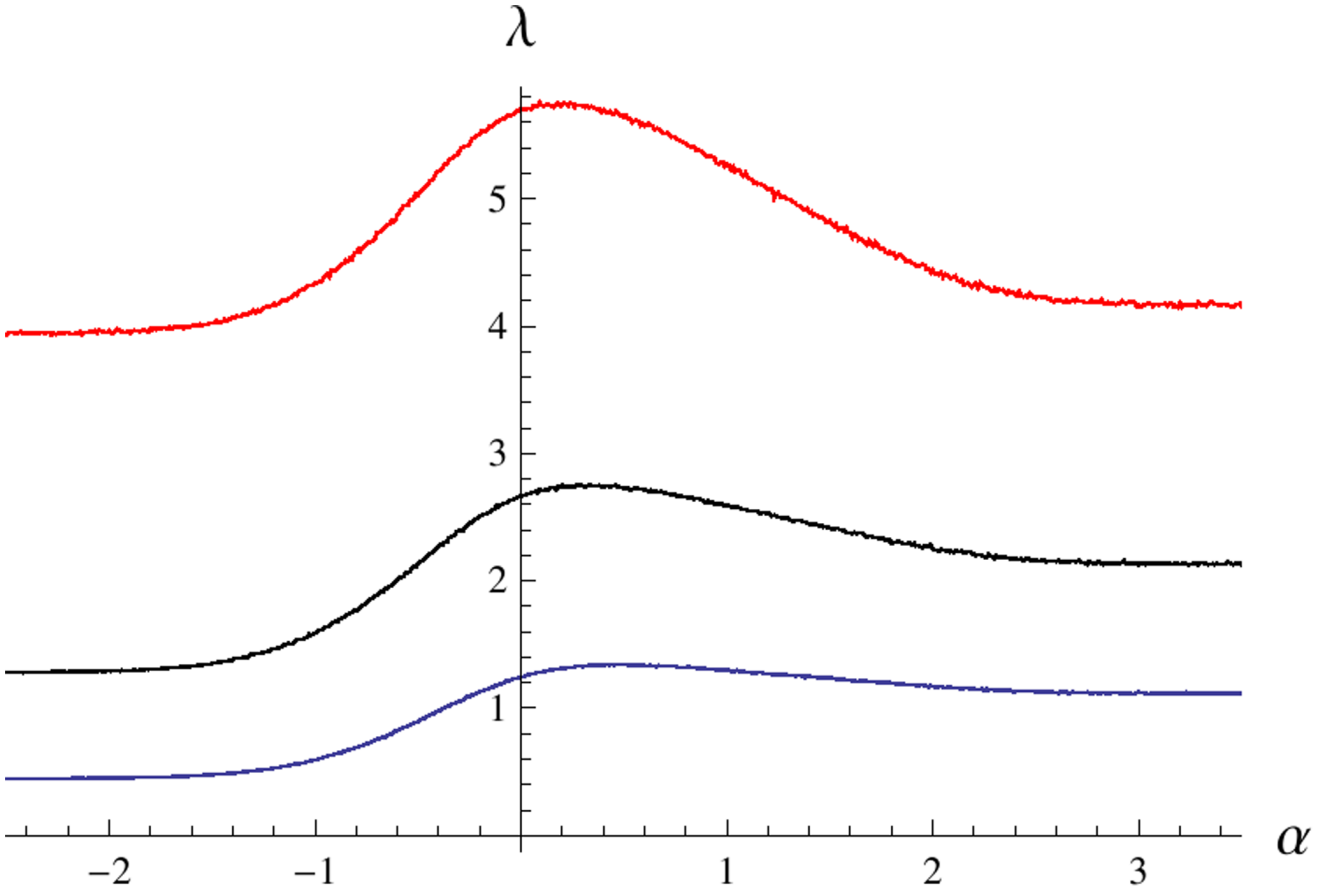
Results of modifying the difference of the growth rates. The parameter 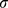 measures the difference of the growth rates of the fully favored and fully unfavored phenotypes. The growth rate 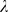 increases with 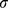. The curves correspond to 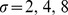. Other parameters are 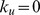, 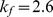, 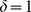, 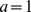 and 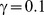.

Simulations of system (7) were performed using the Runge-Kutta method with the input 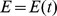 obtained by the Euler method. A typical time step was 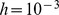; the stochastic term in (4) was modeled by a sequence of independent random variables 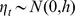. The time interval 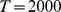 of individual simulations was chosen sufficiently large to ensure the convergence of the growth rate 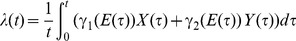
(8)to its asymptotic value (6). Figure 3 presents a typical plot of 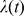 which has two parts corresponding to the first half and the second half of the time interval 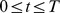. The first part includes the process of relaxation of 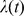 to its stationary value. The second part is almost stationary with deviations from the stationary value within 1%. Since the variance of 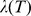 is this small, all the plots presented in Figures 4–6 look almost smooth.

**Figure 7:**
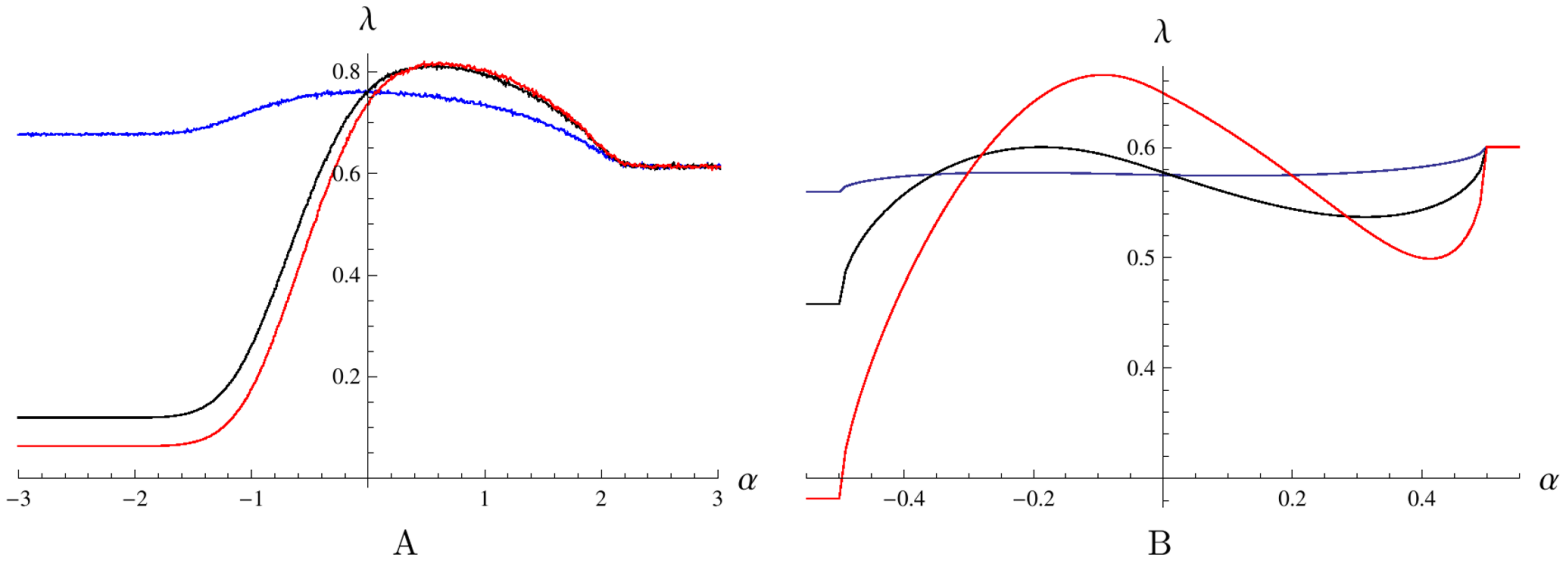
Dependence of the Lyapunov's exponent 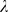 on the parameter 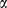 for alternative environmental inputs. A: Results for model (1) with the stochastic input (5). The curves correspond to 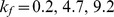. For negative 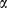 the growth rate 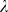 decreases with 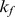. B: Results for model (1) with the periodic environmental input 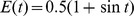. The curves correspond to 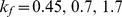. Again, for negative 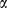 the growth rate 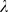 decreases with 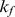. Other parameters are the same as in Figure 4.

Each point of the plots in Figures 4-6 was obtained by averaging the value of 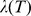 over 20 simulations of system (7) with stochastic input (4). The stepping of 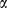 was 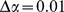. A similar procedure was used to obtain the plots in Figure 7A with the exception that input (4) was replaced by input (5). It has been checked that the plots shown in Figure 4 remain essentially the same when a smaller time step 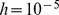 or a longer time interval 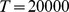 is used.

## Results

The results are presented for zero transition rate 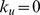 from favored to unfavored phenotype.

When the lag time is close to zero (large 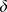), model (1) can be approximated by the system 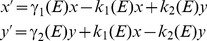
(9)where the non-growing phenotypes have been removed. The results obtained from model (1) with 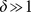 and model (9) are similar. Figure 4A presents the dependence of the average growth rate 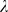 on 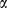 for the system with zero lag phase, which is driven by OU environmental process (4). The horizontal graph corresponds to 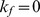, the case of no flow between phenotypes. Here the Lyapunov's exponent 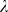 is close to the arithmetic mean 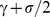 of the saturated highest and lowest growth rates. The other three curves corresponding to positive transition rate 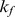 from unfavored to favored phenotype demonstrate a clear maximum, which increases with 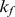. These curves converge to the horizontal graph as 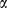 increases, the reason being that for large positive 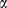 the switching threshold values are so high that the environmental input reaches them rarely, hence little switching occurs and the system behaves almost as in the no flow case 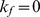. A different behaviour is observed for large in absolute value negative 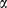. In this limit, the environmental input is unable to pass the threshold values so as to turn off the switching rate, hence each phenotype is constantly transitioning into the other with the effect that the populations are permanently mixing at constant rate 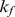. The Lyapunov's exponent 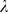 first increases and then decreases with increasing 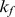 for large in absolute value negative 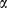.

Figure 4A shows that when the switching rate 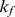 is relatively low, the maximal growth rate 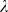 is achieved by the bet-hedging (pessimistic) strategy corresponding to a negative value of 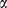, while for larger 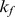 the optimal asymptotic growth occurs for the realistic strategy corresponding to 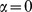. This behavior is consistent with the results of [3] where 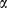 was always zero, but a positive transition rate 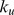 played the role of an (alternative) bet-hedging mechanism. Increasing 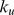 with simultaneously setting a negative 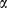 help increase the growth rate in the present model in case of a relatively low switching rate 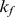 (not shown in the figure).

Figures 4B and 5A illustrate the main finding of this work.

Figure 4B shows that optimal asymptotic growth occurs at positive values of 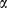 when the lag time 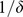 becomes non-zero. A positive 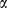 corresponds to the presence of a bi-stability interval which ensures that the cells do not switch phenotype when the environmental variable is placed within the range 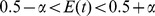. Interestingly, the optimal value of 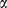 is nearly independent of the maximal switching rates as long as the lag phase delay is the same.

The plot in Figure 4B is divided into three regions, 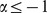 (region 1), 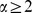 (region 2), and 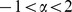 (region 3). The horizontal line corresponding to 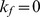, the case where there is no transitions between phenotypes, is the same as in Figure 4A. In region 1, the Lyapunov's exponent 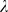 rapidly decreases with 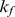. The reason is that the environment is between the thresholds most of the time for this region, 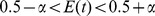, hence the majority of bacteria are nearly always in a transition state due to the pessimistic strategy (negative 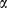). The higher the transition rate 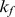, the higher the fraction of the total population that is stuck in the groups 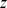 and 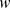 that do not contribute to the growth of the system, hence lower 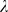. In region 2, the value of 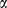 is also sufficiently large so that the environmental input mostly remains within the bi-stability interval. As 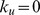, both rates 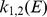 are nearly always zero due to the optimistic strategy corresponding to positive 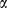 in this region, hence the majority of bacteria are in the non-transition states 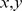 and the plots of 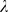 for all 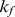 tend to the horizontal plot obtained for 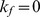 as 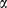 increases. Central Region 3 is the most interesting as each plot 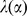 corresponding to a non-zero value of 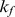 achieves a distinct global maximum at some positive value of 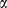, that is for the optimistic switching strategy.

Now, we consider how system (1) responds to variations of parameters. Figure 5 illustrates dependence of positive value of 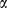 needed to obtain maximum asymptotic growth on the on the lag time and on the “stiffness” parameter of the OU process and. Figure 5A shows that, as the lag time 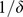 increases, the optimal positive value of 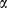 which grants the maximal Lyapunov exponent also increases. There is a direct relationship between 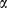 and the average time 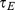 required for the OU process to pass through the bi-stability interval. Hence, the exit time 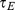 required to optimize the asymptotic growth tunes with the lag time: 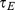 increases with the increasing lag phase delay. This trend is also in agreement with Figure 4A presenting the limit case of zero lag time where the corresponding optimal 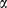 is zero or negative.

Examining the plots showing the dependence 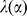 in Figure 5B for several values of the stiffness of the potential well of the environmental input, 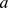, we see that as the well becomes steeper and the environmental input is forced to spend more time around the point 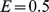 of equal favoring of the phenotypes, the value of the peak in the Lyapunov's exponent 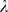 decreases. When the well becomes sufficiently steep, the peak is lost and the Lyapunov's exponent converges to the value 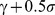 of the average growth rate of the system with no transitions between phenotypes.

In Figure 6, we vary the parameter 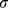, which controls the benefit to the growth rate that bacteria in a favored phenotype gain over bacteria in the unfavored phenotype. Increasing the value of 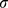 has an effect similar to that of shortening the lag time by increasing 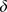, cf. Figure 5A. This result can be understood if we consider 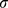 as a penalty for being in the wrong phenotype when the environment changes. When the penalty becomes too high it is no longer worth delaying changing phenotype and becomes better to change with the environment using the realistic switching strategy, that is setting 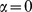.

Finally, we test system (1) with environmental inputs different from the OU process. Figure 7A presents data obtained for input (5) generated by the diffusion process in a double well potential. When the transitions rate 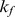 is low, the Lyapunov's exponent is maximized by 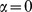. However, as the transition rate 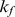 increases, the peak in the Lyapunov's exponent profile 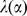 shifts to the region of positive 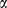. That is, as in case of environmental input (4) (see Figure 4B), the optimistic strategy grants more fitness to the population than the realistic strategy for non-zero lag times.

Plots in Figure 7B were obtained for a periodic input, which represents a fully predictable deterministic pattern of environment variations. Here the graph 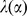 follows a complex profile as 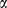 is varied from the region 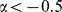, where the transition rates 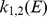 are always equal to 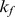, to the region 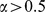, where there are no transitions between the phenotypes (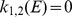). The average growth rate 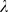 has a local peak in the region 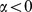 and the peak value increases with 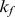. For small transition rates 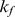, the value of 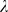 at this local peak is still less than the growth rate 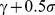, which is achieved for 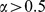 by the regime without transitions. For larger 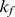, this peak becomes the global maximum, that is the maximum average growth rate is achieved by the bet-hedging (pessimistic) strategy corresponding to an 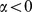. As 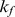 increases further, the peak shifts towards the point 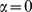. This behavior agrees with the results of [3]. However, we see that for the present model 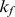 should be large enough to favor the bet-hedging strategy; otherwise, the negative effect of the switching cost dominates and the strategy forbidding transitions between the phenotypes becomes optimal.

Importantly, in case of the periodic environmental input, positive values of 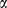 do not help growth for any sufficiently high switching rate 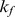. This contrasts with our results for the stochastic environments. In the following discussion, we associate this difference with the fact that the time required for the periodic input to pass through the bi-stability interval does not depend on 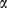.

## Discussion

For the case of zero lag phase, the results obtained in this work are entirely consistent with the results obtained in [3], [4] where only binary environmental signal was considered. The fact that environmental signal is not binary in this work does not appear to have any significant impact on the resulting conclusions. For high switching rate capacity 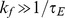 we still conclude that no switching into unfavored phenotype is needed to hedge the bets because the population is capable of adjusting quickly to the environment. For sufficiently low switching rate capacity 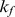 the population is no longer capable of adjusting sufficiently quickly and bet hedging develops through non-zero switching probability into the unfavored state (the optimal switching rate 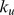 becomes positive). Interestingly, negative threshold separation 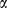, which effectively slows down switching into the favored phenotype in a certain range of the environmental input, can further increase the asymptotic growth rate. This could be viewed as an additional mechanism to tune the characteristic time of phenotype variation to the characteristic time of environmental variations. However, the authors were not able to find any experimental work suggesting such behavior actually occurs perhaps due to the fact that non-zero lag phase is nearly universal among micro-organisms.

The main finding of this paper is that, as Figure 4B illustrates, when the lag phase delays the growth of any phenotype, the optimal phenotype switching strategy involves evolution of a positive threshold separation 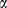. As discussed above, positive threshold separation 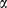 leads to hysteresis for relatively large values of phenotype switching rate 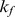 or to nearly hysteretic behavior for smaller values of 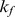.

Experimentally this hysteresis is observed when the environmental variable changes in time slowly and hence phenotype switching occurs at two distinctly different threshold values of the environmental state. On the other hand, at the environmental variation rate for which a particular choice of 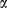 maximizes the asymptotic growth rate, one would not observe hysteresis because short-term switching dynamics is not negligible. In fact, optimal choice of 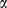 corresponds to a form of stochastic resonance where the delay time associated with the lag phase is a fraction of the average period of the random phenotype switching caused by the random environmental input oscillation. Indeed, model (1) demonstrates the growth of the optimal threshold difference with increasing lag phase time 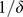, which implies tuning of the average time interval between the switches 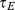 with the lag phase delay 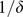. This tuning suggests that measurements of hysteresis and lag phase can help characterize fluctuations of the natural environment. The growth maximizing relationship between the first passage time 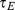 and the lag phase delay time also depends on the effective difference in the growth rates of the two phenotypes.

It is worth noting that deterministic environmental input does not lead to the same type of phenomenon, as illustrated by Figure 1B where the time interval between changes of phenotype switching rates is independent of the threshold difference and is always equal to half the period. This is interesting because it suggests that threshold difference is only useful in the presence of environmental uncertainty helping the system to minimize the risk of changing its phenotype switching rate too often.

The presence of hysteresis in model (1) becomes apparent when the typical rate 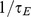 of the input variations is low compared to the inverse lag phase time 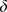 and the rate 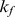 of switching to the favored phenotype but high (due to the low level of noise in the switching thresholds, see Figure 2) compared to the rate 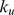 of switches to the unfavored phenotype. These conditions ensure that all bacteria switch quickly and almost simultaneously to the favored phenotype whenever the environmental input leaves the bi-stability interval 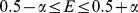 from either end, while there are almost no transitions to the other phenotypes when the environmental input is inside this interval. This is exactly the behavior described by the bi-stable relay illustrated in Figure 1, which represents the simplest form of hysteresis and hysteretic memory. That is, for slow variations of the environmental input, model (1) demonstrates the same memory on the level of the whole population as we assumed in individual bacteria, the reason being that no interaction between organisms has been explicitly included in the model and the lag phase delay as well as separation of the switching thresholds are properties of an individual organism.

The mechanism that promotes the separation of thresholds and its correlation with the rate of environmental variations and the lag phase time can be explained by a trade off between too much responsiveness to environmental variations (for small 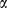), with the associated cost of often transitions, and too much inertia (for large large 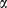), which leaves too many bacteria in unfavored states. The positive threshold difference 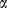 decreases the rate of transitions from less to more favored phenotype when favoring is not strong. Such suppression of back and forth switching agrees with some experimental findings [42], [43]. A slight decrease in the growth rate due to small fluctuations of the environment from the point where both phenotypes are equally favored can be less dramatic than a drop in the growth rate due to passing through the lag phase induced by a switching event.
